# Queen pheromones in *Temnothorax *ants: control or honest signal?

**DOI:** 10.1186/1471-2148-11-55

**Published:** 2011-03-01

**Authors:** Elisabeth Brunner, Johannes Kroiss, Andreas Trindl, Jürgen Heinze

**Affiliations:** 1 LS Biologie I, Universität Regensburg, 93040 Regensburg, Germany; 2 Max Planck Institute for Chemical Ecology, 07745 Jena, Germany

## Abstract

**Background:**

The division of reproductive labor among group members in insect societies is regulated by "queen pheromones". However, it remains controversial whether these are manipulative, i.e., actively suppress worker reproduction, or honestly signal the fertility status of the queen to which workers react in their own interest by refraining from laying eggs. Manipulative queen control is thought to lead to an evolutionary arms race between queens and workers, resulting in complex queen bouquets that diverge strongly among different populations and species. In contrast, honest signals would evolve more slowly and might therefore differ less strongly within and among species.

**Results:**

We aimed at determining the tempo of the evolution of queen signals in two ways. First, we investigated whether queens of *Temnothorax *ants are capable of controlling egg laying by workers of their own, closely, and distantly related species. Second, we compared the species- and caste-specific patterns of cuticular hydrocarbons, which are assumed to convey information on reproductive status. In mixed-species colonies, queens were not able to fully suppress egg-laying and male production by workers of unrelated species, while workers did not reproduce under the influence of a queen from their own species. Furthermore, the chemical profiles differed more strongly among queens of different species than among the respective workers.

**Conclusions:**

Our results suggest that cuticular hydrocarbons associated with fecundity are not fully conserved in evolution and evolve slightly faster than worker-specific components in the blend of cuticular hydrocarbons. While this higher rate of evolution might reflect an arms race between queens and workers, the observation that workers still respond to the presence of a queen from another species support the honest signal hypothesis. Future studies need to examine alternative explanations for a higher rate of evolution of queen-specific substances, such as an involvement of such compounds in mating.

## Background

The efficiency and integrity of the societies of ants, bees, and wasps relies on a well-controlled division of reproduction [1,2]. Workers rarely lay eggs in the presence of a fertile queen [3,4]. This is surprising, as workers are more closely related to their own sons (r = 0.5) than to the sons of the queen (r = 0.25) and in queenless conditions are usually capable of producing male offspring from their own, unfertilized eggs [5,6].

Complete worker sterility benefits the queen, which should be selected to inhibit worker reproduction. However, overt aggression by the queen is very rare and appears restricted to very small colonies [7-10]. Instead, reproduction appears to be controlled chemically by glandular or cuticular pheromones [11,12]. In honeybees, secretions from the mandibular glands and other sources are thought to regulate egg laying in the hive [reviewed in [13,14]]. In contrast, the chemical composition of cuticular waxes is correlated with fecundity in a wide and diverse range of species of ants. This suggests the involvement of cuticular hydrocarbons in the regulation of reproduction [11,12], and indeed, 3-methyl-hentriacontane has recently been shown to regulate worker sterility in the ant *Lasius niger *[15].

The question why workers respond to queen pheromones by foregoing their own reproduction is a special case of the more general, fundamental problem of whether intraspecific communication is honest or manipulative. Queen-specific chemicals might act as primer pheromones that actively suppress the ovaries of workers [16,17]. However, it has been argued that such inhibitive queen control were instable in evolution if acting against the fitness interests of the workers [18]. Mutations rendering workers insensitive to queen inhibition would spread in the population, again changing the selection pressures on queens and favoring queen mutations that qualitatively or quantitatively changed their manipulative agents. The resulting arms race between queens and workers would eventually lead to more and more complex pheromone mixtures. As an alternative, pheromones produced by the queens might honestly signal their level of fertility. Workers might respond in their own interest, e.g., to avoid being attacked ("policed") by the queen or other workers [11,18-21]. Worker altruism might therefore be "enforced" [21-25].

Honest signaling requires that the quantity or quality of queen pheromones is strictly associated with their level of fertility and mating status [18]. This, however, is often not the case. For example, unmated reproductives may produce similar pheromonal bouquets as mated reproductives [11,22]. Furthermore, the hypothesis of manipulative regulation of reproduction appears to gain renewed support from the observation that, at least in honeybees, the regulation of reproduction is based on a very large number of substances from multiple glands [13,14].

Distinguishing between queen control and honest signaling is difficult without detailed knowledge about the molecular and cellular mechanisms involved. However, both mechanisms might leave different traces in evolution [11]. The series of manipulation and counter-manipulation associated with the scenario of queen control results in a rapid evolution of queen compounds. Queen pheromones therefore likely differ even between related species. In contrast, honest signals are expected to be more stable in evolution and to evolve more slowly. Unfortunately, little is known about the variation of fertility-associated chemical compounds among related species. In this study, we compared the composition of worker and queen cuticular hydrocarbons among different species of the ant genus *Temnothorax*. In addition, we investigated whether workers begin laying eggs in the presence of a queen from another, closely or distantly related species. We hypothesized that with queen control and rapid evolution, queens should be less efficient in suppressing ovary development by workers from another species. Furthermore, we expected the chemical bouquet of queens to differ more between species than those of workers. In contrast, in the case of honest signaling, workers would react to fertility signals by queens from another species, and the bouquets of queens from different species would be not more different than those of workers.

## Results

### Worker ovary activation and male-production by workers

**Figure 1:**
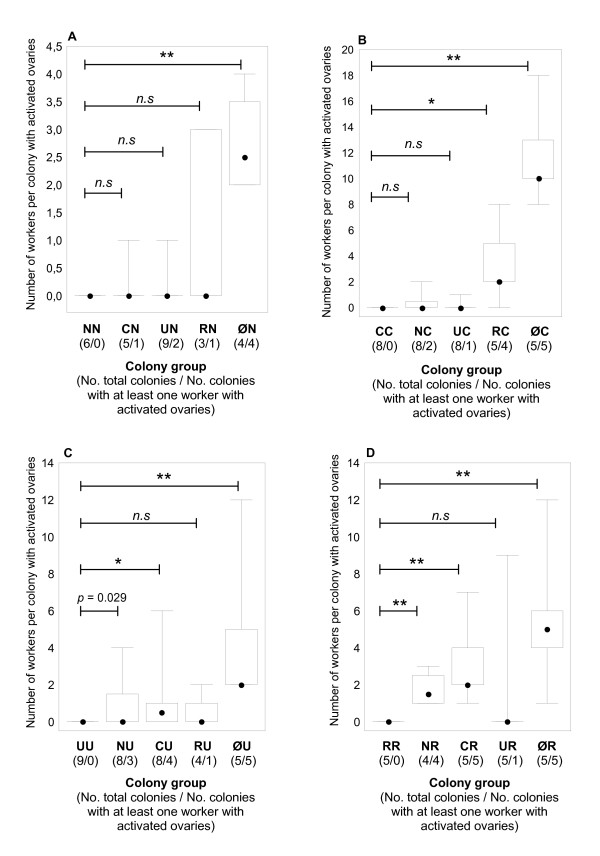
**a-d - Worker ovary activation in mixed species colonies of *Temnothorax *ants**. Number of workers per colony with activated ovaries. Minimum and maximum (horizontal line*s*), first and third quartiles (rectangle), and the median (dot) are shown. The total number of colonies and the number of colonies with at least one worker with activated ovaries in each group is given in parentheses. *P*-values are from two-sample permutation tests (*p < 0.01; **p < 0.0001; n.s. nonsignificant). After Bonferroni's correction, *p*-values of < 0.01 are significant at the 0.05 level. **1a**. *T. nylanderi *workers in colonies with a *T. nylanderi *queen (NN, control), *T. crassispinus *queen (CN), *T. unifasciatus *queen (UN), *T. recedens *queen (RN) and in colonies without a queen (ØN). **1b**. *T. crassispinus *workers in colonies with a *T. crassispinus *queen (CC, control), *T. nylanderi *queen (NC), *T. unifasciatus *queen (UC), *T. recedens *queen (RC) and in colonies without a queen (ØC). **1c**. *T. unifasciatus *workers in colonies with a *T. unifasciatus *queen (UU, control), *T. nylanderi *queen (NU), *T. crassispinus *queen (CU), *T. recedens *queen (RU) and in colonies without a queen (ØU). **1d**. *T. recedens *workers in colonies with a *T. recedens *queen (RR, control), *T. nylanderi *queen (NR), *T. crassispinus *queen (CR), *T. unifasciatus *queen (UR) and in colonies without a queen (ØR).

Experimental colonies, in which queens and workers either belonged to the same species or to different species, were created by exchanging worker pupae among colonies. Ovary dissection showed that all queens had elongated ovaries with numerous yellow bodies and maturing eggs, i.e., they were fully fertile. None of the workers in the control colonies had activated ovaries (colony groups NN, CC, UU, and RR; Figure 1a-d). Though we had observed sporadic worker egg-laying in queenright colonies of *T. recedens *in a previous study (unpublished data), dissection data did not corroborate this result for the presently studied colonies.

In all queenless colonies, the ovaries of several workers were activated (Figure 1a-d). This indicates that workers are capable of activating their ovaries within six weeks after removal of the queen. Fertile workers were also found in some of the mixed-species colonies, such as RC (Figure 1b), CU (Figure 1c), NR and CR (Figure 1d; for colony abbreviation see Table 1).

**Table 1:** Mixed-species colony set ups composed of four different *Temnothorax *species.

Colony composition		Colony name	Colonies set up in 2005	Colonies set up in 2006	No. total colonies	No. colonies used for the assessment of ovary activation	No. colonies used for the assessment of worker male-production
Queen species	Worker species						
*T. nylanderi *(control)	*T. nylanderi*	NN	NN1 - NN5	NN6 - NN15	15	10	5
*T. crassispinus*		CN	CN1 - CN5	CN6 - CN15	15	10	5
*T. unifasciatus*		UN	UN1 - UN5	UN6 - UN15	15	10	5
*T. recedens*		RN		RN1 - RN10	10	5	5
no queen		ØN		ØN1 - ØN5	5	5	-

*T. crassispinus *(control)	*T. crassispinus*	CC	CC1 - CC5	CC6 - CC15	15	10	5
*T. nylanderi*		NC	NC1-NC5	NC6-NC15	15	10	5
*T. unifasciatus*		UC	UC1 - UN5	UC6 - UN15	15	10	5
*T. recedens*		RC		RC1 - RC10	10	5	5
no queen		ØC		ØC1 - ØC5	5	5	-

*T. unifasciatus *(control)	*T. unifasciatus*	UU	UU1 - UU5	UU6 - UU15	15	10	5
*T. nylanderi*		NU	NU1-NU5	NU6-NU15	15	10	5
*T. crassispinus*		CU	CU1 - CU5	CU6 - CU15	15	10	5
*T. recedens*		RU		RU1 - RU10	10	5	5
no queen		ØU		ØU1 - ØU5	5	5	-

*T. recedens *(control)	*T. recedens*	RR		RR1 - RR10	10	5	5
*T. nylanderi*		NR		NR1 - NR5	5	5	-
*T. crassispinus*		CR		CR1 - CR5	5	5	-
*T. unifasciatus*		UR		UR1 - UR5	5	5	-
no queen		ØR		ØR1 - ØR5	5	5	-

Total no. of colonies:			45	165	210	145	65

Colonies were set up in the years 2005 and 2006. The number of colonies randomly chosen for the assessment of ovary activation and for the assessment of worker male-production is given. The first letter in colony denominations indicates the species of the queen (Ø for queenless), the second the species of the workers.

In 2007, a total of 752 males were collected from 60 colonies (Table 2). Five colonies, in which the queen or most of the workers had died, were excluded from further analyses. In all colonies, except RC8, queens had fully developed and activated ovaries with yellow bodies and maturing eggs.

**Table 2:** Male production by queens and workers in control and mixed-species colonies composed of different *Temnothorax *species.

Colony	No. total males produced per colony	No. males *Allozyme electromorph*	No. males produced by the queen	No. males produced by workers
NN11, NN13, NN14, NN15	none			
CN7, CN12	none			
CN8	15	15 *f*	-	15
CN13	3	3 *m*	3	-

UN7, UN11, UN12, UN14, UN15	none			

RN4	3		3	-
RN5	20		11	9
RN6	20		15	5
RN8	5		5	-

CC12, CC13, CC14, CC15	none			

NC11, NC12, NC13, NC14, NC15	none			

UC7, UC13, UC15	none			
UC6	3	1*s*, 2*m*	1	2
UC8	1	1*m*	-	1

RC3	6		6	-
RC4	2		2	-
RC6	1		1	-
RC8	54		1	53*

UU6	1		1	-
UU8	2		2	-
UU9	1		1	-
UU10	5		5	-
UU12	29		29	-

NU10	8	8*s*	-	8
NU12	74	42*s*, 19*m*, 13*f*	13	61
NU13	37	28*s*, 3*m*, 6*f*	6	31
NU14	13	6*s*, 7*f*	7	6
NU15	59	59*s*	-	59

CU6	8	8*s*	-	8
CU9	2	2*s*	-	2
CU12	14	6*s*, 8*m*	-	14
CU15	44	26*s*, 18*m*	18	26

RU7	31		31	-
RU8	95		27	68
RU9	105		105	-

RR1	27		27	-
RR4	10		10	-
RR5	7		7	-
RR6	8		8	-
RR8	24		24	-

*In colony RC8 the ovaries of the queen were not fully developed.

Numbers of males produced per colony by the queen or by workers are shown, respectively. Colony composition and colony names are explained in Table 1. Explanation of Allozyme electromorphs are given in the text.

Allozyme analyses revealed that males in mixed-species colonies were produced by the queen (e.g., CN13), by the workers (e.g., CN8) or both (e.g., RN5, NU12, CU15, Table 2). Queens of colonies UC6 and UC8 were homozygous for the *s *allele, and *m-*males were therefore offspring of *T. crassispinus *workers, while the one *s-*male was son of a *T. unifasciatus *queen. All queens of colonies NU10 to NU15 were homozygous *ff*, and the *s- *and *m*-males were therefore offspring of *T. unifasciatus *workers.

In the CU colonies, *s*-males were presumably produced by *T. unifasciatus *workers, but *m-*males could in principle be sons of a *T. crassispinus *queen or *T. unifasciatus *workers. Mitochondrial DNA analysis revealed that the eight males in colony CU12 were offspring of *T. unifasciatus *workers, while the 18 males from the colony CU15 were produced by the *T. crassispinus *queen.

### Cuticular hydrocarbons of queens and workers from different species of *Temnothorax *ants

The cuticular profiles of queens and workers of the six *Temnothorax *species consisted of a total of 64 peaks (Numbers of peaks in each species is given in Table 3). 40 peaks consistently appeared in all six species and 47 peaks could be identified by GC-MS [Additional file 1. Representative chromatography profiles from a queen of each species; Additional file 2. Proportions (%) of peak areas from cuticular hydrocarbon extracts of queens and workers of each species; Additional file 3. Identification of cuticular compounds and differences of their relative amounts between queens (Q) and workers (W) of each species; see also [26] for identification of compounds in *T. unifasciatus*]. The profiles of queens and workers were predominantly characterized by the linear alkane *n*-C_27 _(Additional file 1, 2), while individuals of *T. recedens *were characterized by several longer chained hydrocarbons. Due to their very low abundance, peaks 36 to 40 and a few other peaks marked in Table S1 (Additional file 3) with an asterisk could not be identified. However, the latter peaks had exactly the same retention time as peaks in other species, which could be identified and are therefore assumed to be chemically identical to these compounds.

**Table 3:** Discriminant Analysis between groups of queens and workers within six *Temnothorax *species based on their specific cuticular hydrocarbon profiles.

Species	No. of peaks	Wilks' λ	F-values	*p*-levels	correct classification
*T. nylanderi*	46	0.1098	F _(7.7) _= 8.105	< 0.01	100%
*T. crassispinus*	48	0.0068	F _(10.4) _= 58.828	< 0.001	100%
*T. unifasciatus*	46	0.0614	F _(8.8) _= 15.290	< 0.0001	100%
*T. recedens*	52	0.1919	F _(11.32) _= 12.248	< 0.0001	100%
*T. lichtensteini*	48	0.1326	F _(8.14) _= 11.453	< 0.0001	100%
*T. affinis*	46	0.1972	F _(8.13) _= 6.616	< 0.01	100%

Percentages of correct classifications of individuals from predefined groups are given in the right column.

**Figure 2:**
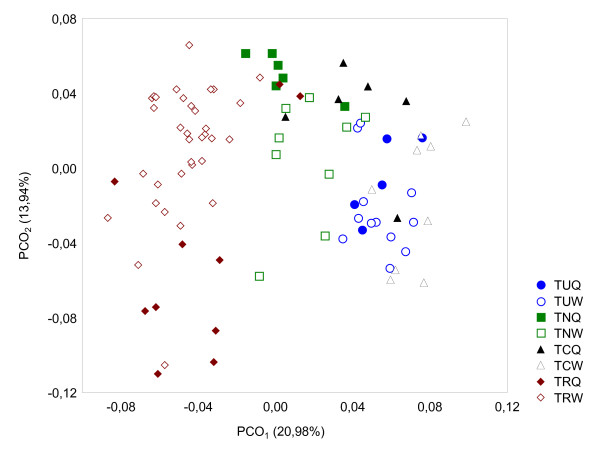
**PCO ordination based on the cuticular hydrocarbons profiles from four *Temnothorax *species**. Bidimensional PCO ordination based on the cuticular hydrocarbons profiles of queens (Q) and workers (W) from four different species of *Temnothorax *ants involved in the mixed-species experiment: *T. unifasciatus *(*Tu *Q, *n *= 5; *Tu *W, *n *= 12), *T. nylanderi *(*Tn *Q, *n *= 6; *Tn *W; *n *= 9), *T. crassispinus *(*Tc *Q, *n *= 6; *Tc *W, *n *= 9), *T. recedens *(*Tr *Q, *n *= 10; *Tr *W; *n *= 34). The percentages of variance explained by the two main principal coordinates are given in parentheses.

The chemical distances between *T. nylanderi *and *T. crassispinus *queens are lower compared to the other pair-wise comparisons among queens (Figure 2; Table 4). The groups of *T. recedens *queens and workers are chemically most distant from the groups of the other three *Temnothorax *species (Figure 2; Table 4). Interestingly, distances among queens are higher than among workers in four of six pair-wise comparisons (Table 4).

**Table 4:** Chemical distances between queens and workers among four *Temnothorax *species.

Queens	*Tn *Q	*Tc *Q	*Tu *Q	Workers	*Tn *W	*Tc *W	*Tu *W
***Tc*****Q**	**0.077**			***Tc*****W**	0.093		
***Tu*****Q**	0.108	0.088		***Tu*****W**	0.093	**0.066**	
***Tr*****Q**	0.116	0.125	0.120	***Tr*****W**	0.093	0.128	0.110

Euclidean resemblance matrix based on cuticular hydrocarbon profiles of queens (Q; left side) and workers (W; right side) from the four species of *Temnothorax *ants involved in the mixed-species experiment (*Tn *= *T. nylanderi*, *Tc *= *T. crassispinus*, *Tu *= *T. unifasciatus*, *Tr *= *T. recedens*). Figures in bold indicate a close Euclidean Distance between a pair of groups in contrast to the other compared pairs of groups.

Within each species, chemical distances between groups of queens and workers are statistically significant and their cuticular profiles are classified correctly in a discriminant analysis (Table 3).

## Discussion and Conclusions

Our study about the cross-specificity of the chemical compounds used in the regulation of reproduction in colonies of *Temnothorax *ants reveals a promising new approach to answering the question of whether queen pheromones are manipulative or honest signals. Queen pheromones appear to be less active across species-borders than within species. No worker reproduction was observed in single-species colonies. In contrast, queens were not able to fully prevent ovary development and male-production by workers from non-related species, which indicates that queen pheromones are not fully conserved in evolution in this group of species. Furthermore, the rate of worker reproduction and ovary development of workers in mixed-species was lower than in queenless colonies, which speaks against the rapid evolution of queen pheromones expected from the queen control hypothesis.

These behavioral results are reflected in the chemical profiles of queens and workers, which in most pair-wise comparisons differed more between queens than between workers from different species but not tremendously so. This might suggest that queen bouquets diverge slightly more quickly than those of workers, but not at an extremely rapid speed. A faster divergence of queen- than worker-specific substances is in agreement with the hypothesis of a queen-worker arms race with manipulation and counter-manipulation. However, it might also reflect other forces of selection. For example, if queen-specific cuticular compounds were involved in attracting mates, avoidance of hybridization would quickly lead to species-specific queen bouquets. More information about the contexts in which cuticular substances are used in communication is therefore needed.

Studies on the paper wasp *Polistes dominulus *suggested a relatively high speed of evolution. Here, the cuticular hydrocarbons that differed between egg layers and non-reproductives varied even between different populations [27]. The observation that the chemical profiles of *T. crassispinus *and *T. nylanderi *queens are more similar compared to the other *Temnothorax *species, even though the two taxa diverged more than 1 Million years ago [28], makes similar intraspecific variation unlikely, but this remains to be investigated.

Our data do not reveal a clear trend in the chemistry of those hydrocarbons that differentiate queens and workers among the six investigated species. This matches the heterogeneous picture found in other, less closely related ants, where reproductives are characterized by particular long-chained hydrocarbons in some species but shorter or branched hydrocarbons in others [12,15,29-31]. However, it needs to be pointed out that it is usually not known, which of the large number of substances that differ between queens and workers are biologically active and which are mere side-products of the hydrocarbon metabolism without a function in communication [11].

Our study reveals a number of species idiosyncrasies that do not match phylogenetic relationships and are difficult to explain in the light of hypotheses about the nature of queen pheromones. For example, *T. nylanderi *and *T. crassispinus *workers seem to respond to the presence of a *T. unifasciatus *queen, while *T. unifasciatus *workers readily develop their ovaries in the presence of a *T. nylanderi *or *T. crassispinus *queen. This resembles the situation in honeybees, in which the presence of a heterospecific queen increases the rate of worker ovary activation strongly in *Apis cerana *but only slightly in *Apis mellifera *[32]. In contrast, workers of the bumblebee *Bombus terrestris *develop their ovaries in queenright colonies of the phylogenetically related *B. lapidarius *at a similar rate as under queenless conditions, but not in queenright homospecific colonies [33]. The results in these studies might have been affected by the presence of workers belonging to the species of the queen, as they might have an interest in preventing heterospecific workers from reproducing through aggression or egg eating. Worker nepotism might explain the common absence of reproduction by host workers in socially parasitic ants. *Temnothorax *workers are often parasitized by queens of slave-making species, such as *Chalepoxenus*, *Myrmoxenus *or *Protomognathus*. Though these genera are less closely related to *Temnothorax *than the pairs of species used in our study [e.g., [34]], enslaved workers rarely produce males [e.g., [35-37]]. Either slave-making queens have evolved particularly manipulative queen pheromones, which are active across large phylogenetic distances, or their reproductive monopoly is additionally enforced by aggression. Indeed, both queens and workers of slave-making ants have been observed to attack host workers in a way resembling the dominance interactions among the slave-makers themselves [8,37].

Though our results do not allow drawing final conclusions about the speed of queen pheromone evolution, comparisons of their cross-species activity might help to learn more about the nature of such pheromones and how quickly they diverge among species. The heterogeneity of queen substances might reflect an ongoing arms race between queens and workers [14]. However, it might also be a consequence of species-specific queen-bouquets serving to avoid hybridization. The fact that workers in mixed-species colonies did not behave like workers in queenless colonies strongly suggests that workers recognize queens belonging to another species and that queen pheromones regulating worker reproduction are partly conserved in evolution. This would be in agreement with the honest signal hypothesis.

## Methods

In spring 2005 and 2006 we collected complete colonies of six different *Temnothorax *species: *T. nylanderi *(Förster, 1850) and *T. affinis *(Mayr, 1855) in Sommerhausen (Würzburg, Germany), *T. crassispinus *(Karavejev, 1926) in Unterisling (Regensburg, Germany), *T. unifasciatus *(Latreille, 1798) in Waldenhausen (Germany) and Gargnano (Lago di Garda, Italy) and *T. recedens *(Nylander, 1856) and *T. lichtensteini *(Bondroit, 1918) Gargnano (Lago di Garda, Italy). While *T. nylanderi *and *T. crassispinus *are closely related sibling species [38], the other four species are phylogenetically more distantly related (Additional file 4).

*Temnothorax *colonies were collected from their nests in decaying branches on the ground and, in *T. unifasciatus*, *T. lichtensteini *and *T. recedens*, also from crevices in stone walls. The colonies were transferred into small plastic boxes (10 cm × 10 cm × 3 cm) with a regularly moistened plaster floor and kept in incubators under artificial climate conditions with the temperature gradually being raised from spring (10°C night/20°C day) to summer (17°C night/28°C day) conditions [39,40]. Twice per week, colonies were provided with water, honey, and pieces of cockroaches.

### Mixed-species colony set up

In 2005, colonies of *T. nylanderi*, *T. crassispinus *and *T. unifasciatus*, with a sufficient amount of larvae in each colony, were chosen for the mixed-species experiment (*N *= 45 colonies; Table 1). In 2006, the same mixed-species colonies were set up with additional mixed colonies plus *T. recedens *and colonies without a queen (*N *= 165 colonies; Table 1). The number of worker pupae in *T. recedens *colonies was restricted; therefore, only five mixed colonies with *T. recedens *worker pupae were set up (Table 1). Mixed colonies were set up in early summer, when most larvae had developed into pupae. We transferred 50 worker pupae of the same species into a nest with either a queen from a different species in mixed colonies or a con-specific queen in control colonies (Table 1). To obtain the required sample size of 50 pupae, worker pupae were taken from five different con-specific colonies. No larvae or eggs were added to the colonies. To allow worker pupae to fully develop, we placed 30 marked adult workers from the colony of queen origin into each nest and removed them four weeks later after most of the pupae had developed into adult workers.

Several experimental colonies, in which the transferred worker pupae did not develop into adults (2005: 18 of 45 colonies; 2006, 8 of 100 colonies), had to be excluded from the study.

### Worker ovary activation

In 2005, worker ovary activation was investigated in all colony set ups. In 2006, we investigated worker ovary activation in all colonies without a queen, all colonies with *T. recedens *workers and a queen from a different species, and five randomly chosen colonies of each of the remaining colony set ups (total *N *= 145 colonies; Table 1). The colonies were frozen six weeks after the transferred worker pupae had developed into adult worker, and workers and queens were dissected to assess their ovary activation [41]. Workers having elongated ovaries (> 1 mm) with viable, oval eggs similar in shape and color to those found in the ovaries of queens were classified as "fertile".

For statistical analyses, two sample permutation tests were used to assess the difference of numbers of fertile workers per colony between groups of control colonies and mixed colonies and between control colonies and colonies without a queen.

### Male-production by workers

The remaining 65 colonies of the 2006 set up were kept in incubators (gradual decrease of temperature to 0°C night/10°C day for 15 weeks and gradual increase again to 17°C night/28°C day thereafter) until hibernated brood had developed in 2007 (Tables 1 and 2). From May to August 2007 all freshly enclosed adult males were collected and frozen at -20°C for further analyses. After all male pupae had enclosed, all colonies were frozen and queens were dissected to determine their ovarian status.

*T. nylanderi*, *T. crassispinus *and *T. unifasciatus *males are of dark brown pigmentation. *T. recedens *males have a pale pigmentation and could easily be distinguished from males of the other three species by inspecting their coloration. *T. nylanderi*, *T. crassispinus *and *T. unifasciatus *males are morphologically similar and thus were distinguished by electrophoresis of the glucose-6-phosoate isomerase [GPI; [27,38]] or sequencing the mitochondrial cytochrome b (Cyt b) gene.

#### Allozyme analyses

Electrophoresis of glucose-6-phosoate isomerase for *Temnothorax *ants has been described previously [27]. Electromorphs were named according to their migration velocities in the gel (fast *f*; medium *m*; slow *s*). *T. crassispinus *and *T. nylanderi *are fixed almost completely for the electromorphs *m *and *f*, respectively [27,38] and *T. nylanderi *occasionally exhibits the electromorph *s *[27]. In *T. unifasciatus*, 32 of 36 workers from 20 colonies were homozygous for the electromorph *s *and 4 were heterozygous with electromorph genotype *sm *[see also [42]]. Queens were analyzed when necessary.

The gasters of individual workers and queens were homogenized in 20 μl Tris-EDTA pH 7.0 buffer. Proteins were separated by 90 min electrophoresis at 10 V/cm and 20 mA on 10 cm × 8 cm × 0.75 mm 7.5% polyacrylamide slab gels using a Tris-glycine pH 8.3 buffer. The enzyme was stained using standard histochemical techniques [43].

#### Mitochondrial analyses

When males could not be distinguished by electrophoresis, we in addition sequenced the cytochrome b (Cyt b) gene. DNA was extracted from the gasters of males using the CTAB method (1%) as previously described [44]. The mitochondrial cytochrome b (Cyt b) gene was analyzed using the primers CbI (CB-J-10933) and 16Sar (LR-N-13398) [45]. The 20 μl PCR reaction mixture consisted of 1 μl DNA, 0.125 mM dNTPs, 0.25 μM of each primer, 11.1 μl dd H_2_O, 2 μl 10× PCR buffer (MBI), 2.5 mM MgCl_2 _and 1 μl of 1 unit/μl Taq Polymerase. Genes were amplified at an annealing temperature of 48°C with 38 cycles. PCR products were separated by electrophoresis on a 1% ethidiumbromide-stained agarose gel (TAE buffer) for 30 min at 100 mA and then purified with High Pure PCR cleanup Micro Kit (Roche). Cycle sequencing was carried out with 3 μl of purified PCR-Product using ABI-Cycle sequencing Kit Version 1.1. Single-stranded PCR products were sequenced using an ABI PRISM 310 automatic sequencer (Perkin-Elmer, Applied Biosystems). The first 450 base pairs of the Sequences representing the Cyt b gene were read and aligned with Sequencing Analysis Software version 3.4 (Perkin-Elmer, Applied Biosystems).

### Cuticular hydrocarbons of queens and workers from different species of *Temnothorax *ants

To estimate the chemical distances between the four species of *Temnothorax *ants used for the mixed-species colony set ups, queens and workers from *T. nylanderi*, *T. crassispinus*, *T. unifasciatus*, and *T. recedens *were analyzed. For the identification of queen specific signals, queens and workers from two additional species, *T. affinis *and *T. lichtensteini*, were included in the analysis. From each species the queens of 5 to 10 unmanipulated colonies plus 1 to 3 workers from each of the colonies were chemically analyzed. All colonies were collected in spring 2006 (see above). *T. unifasciatus *colonies were used only from the population in Italy.

#### Chemical Analysis

Hydrocarbons were extracted four to five weeks after colonies had been subjected to artificial summer condition (17°C night/28°C day; see above). Workers were frozen and hydrocarbons were obtained through solvent extraction by individually immersing each worker for 10 min in 20 μl pentane. After evaporation of the solvent, the residues were re-dissolved in 15 μl pentane, of which 2 μl were injected into an Agilent Technologies 6890N gas chromatograph. Hydrocarbons of queens were obtained through SPME (Solid Phase Micro Extraction) which gives qualitative and quantitative similar results [46]. A 30 μm polydimethylsiloxane fiber was gently rubbed for 10 min against the gaster of the immobilized queen and injected into the injection port of the same gas chromatograph as above. The gas chromatograph was equipped with a flame ionization detector and a HP-5 capillary column (30 m × 0.32 mm × 0.25 μm, J&W Scientific, USA). The injector was split/splitless and the carrying gas was helium at 1 ml/min. The same temperature program was used for the solvent and the solid phase micro extraction with the temperature initially held at 70°C for 1 min, increased from 70°C to 180°C at 30°C/min, from 180°C to 310°C at 5°C/min, and held constant at 310°C for 5 min.

For identification of the peaks, the pooled extracts of 30 workers of each species were injected into a combined gas chromatography and mass spectrometry (GC-MS; Agilent Technologies 6890N) equipped with a RH- 5 ms+ fused silica capillary column (30 m × 0.25 mm × 0.25 μm, J&W Scientific, USA). The injector was split/splitless (250°C) with the purge valve opened after 60 sec and the carrying gas was helium at 1 ml/min. Temperature was held constant for 1 min at 60°C, increased from 60°C to 300°C at 5°C/min and held constant for 10 min at 300°C. The electron impact mass spectra (EI-MS; Agilent 5973 inert mass selective detector) were recorded with an ionization voltage of 70 eV, a source temperature of 230°C and an interface temperature of 315°C. We identified *n*-alkanes by comparing mass spectra with data from a commercial MS library (NIST, Gaithersburg, MD, USA). Methyl-alkanes were identified by diagnostic ions, standard MS databases (see above), and by determining Kovats indices by the method of Carlson et al. [47]. MSD ChemStation Software (Agilent Technologies, Palo Alto, CA, USA) for Windows was used for data acquisition.

For statistical analysis of the chemical distance between the four species involved in the mixed-species experiment, we included peaks consistently present in queens and workers of all four species, plus peaks with a relative area of more than 1% that were present in at least 50% of individuals in a group of workers or queens within each species. Standardized peak areas were transformed by square root. Principle coordinate (PCO) analyses based on Gower's centered matrix was used to visualize the patterns of differences in the multivariate chemical structure among groups [48-50]. Euclidean distance matrix was analyzed based on centroids of groups calculated from principle coordinates. PCO and Euclidean distance analyses were performed using the program PCO [49].

For the identification of queen specific signals we analyzed each species separately and included peaks consistently present in the groups of queens and workers within each species. Standardized peak areas were transformed by using the formula: Z*ij *= log[X*i, j*/g(X*j*)], with X*i, j *being the standardized peak area *i *for the sample *j*, and g(X*j*) the geometric mean of all peaks of the sample *j *[51]. For multivariate analyses, the number of variables was reduced by principle component analysis (PCA). The factor scores obtained by PCA were used in a subsequent discriminant analyses (DA) to determine whether groups could be distinguished on the basis of their cuticular profiles. Wilks' λ significance and the percentage of correct assignments were used to evaluate the validity of the discriminant function. We used Mann-Whitney U-tests to compare percentages of single compounds between groups and adjusted *p*-values for multiple comparisons using Bonferroni's method. PCA and DA analyses were performed using Statistica 6.0.

## Authors' contributions

EB participated in the design of the study, performed all behavioural, morphological, allozyme, chemical, and statistical analyses and participated in writing the manuscript. JK identified the compounds in the chemical analysis. AT carried out the mitochondrial analysis. JH conceived of the study, participated in its design and in writing the manuscript. All authors read and approved the final manuscript.

## Supplementary Material

Additional file 1 **Gas chromatography profiles**. Representative gas chromatography profiles of queens from six *Temnothorax *species. Peaks used for the statistical analysis are marked with numbers. Identification of peaks is given in Additional file 3. Click here for file

Additional file 2 **Proportion of peak areas**. Proportions (%) of peak areas in chromatograms from cuticular hydrocarbon extracts of queens and workers in six *Temnothorax *species. Box plots show medians and 25% and 75% quartiles. Whiskers depict the range of 90% of all cases. Extreme outliers are denoted by circles. *P*-values of substances differing significantly between the various groups are given in Additional file 3. Click here for file

Additional file 3 **Table S1 - Identification of cuticular compounds**. Identification of cuticular compounds and differences of their relative amounts between queens (Q) and workers (W) in five *Temnothorax *species. The number of samples of queens and workers is given in parentheses. Peak numbers correspond with numbers in Additional file 1 and 2. Directions of difference are shown in Additional file 2. Bold *p*-values from Mann-Whitney U-tests are significant at the 5% probability after Bonferroni's correction (*p' *< 0.001); *n.s *= not significant. *Due to very low abundance, peaks marked with a star could not be identified. They had exactly the same retention time in GC as peaks in other species, which could be identified. We therefore assume these compounds to be chemically identical. Click here for file

Additional file 4 **Phylogenetic tree of *Temnothorax *species**. Phylogenetic tree of *Temnothorax *species. Majority rule consensus tree recovered in a Bayesian analysis (4,000,000 generations) with the GTR + I + G model. The tree is based on 651 base pairs of the mitochondrial CO I gene and numbers represent clade credibility values (J. Beibl, pers. comm.). Click here for file
