# Supplementary material for: Queen pheromones in Temnothorax ants: control or honest signal?
Source: BMC Evol Biol. 2011 Mar 1;11:55. doi: 10.1186/1471-2148-11-55 (PMC3060118; doi:10.1186/1471-2148-11-55)

Queen pheromones in *Temnothorax* ants: queen control or honest signal?
Brunner E., Kroiss J., Trindl A. und J. Heinze

Additional file 1 – Gas chromatography profiles

Representative gas chromatography profiles of queens from six *Temnothorax* species. Peaks used for the statistical analysis are marked with numbers. Identification of peaks is given in Additional file 3.

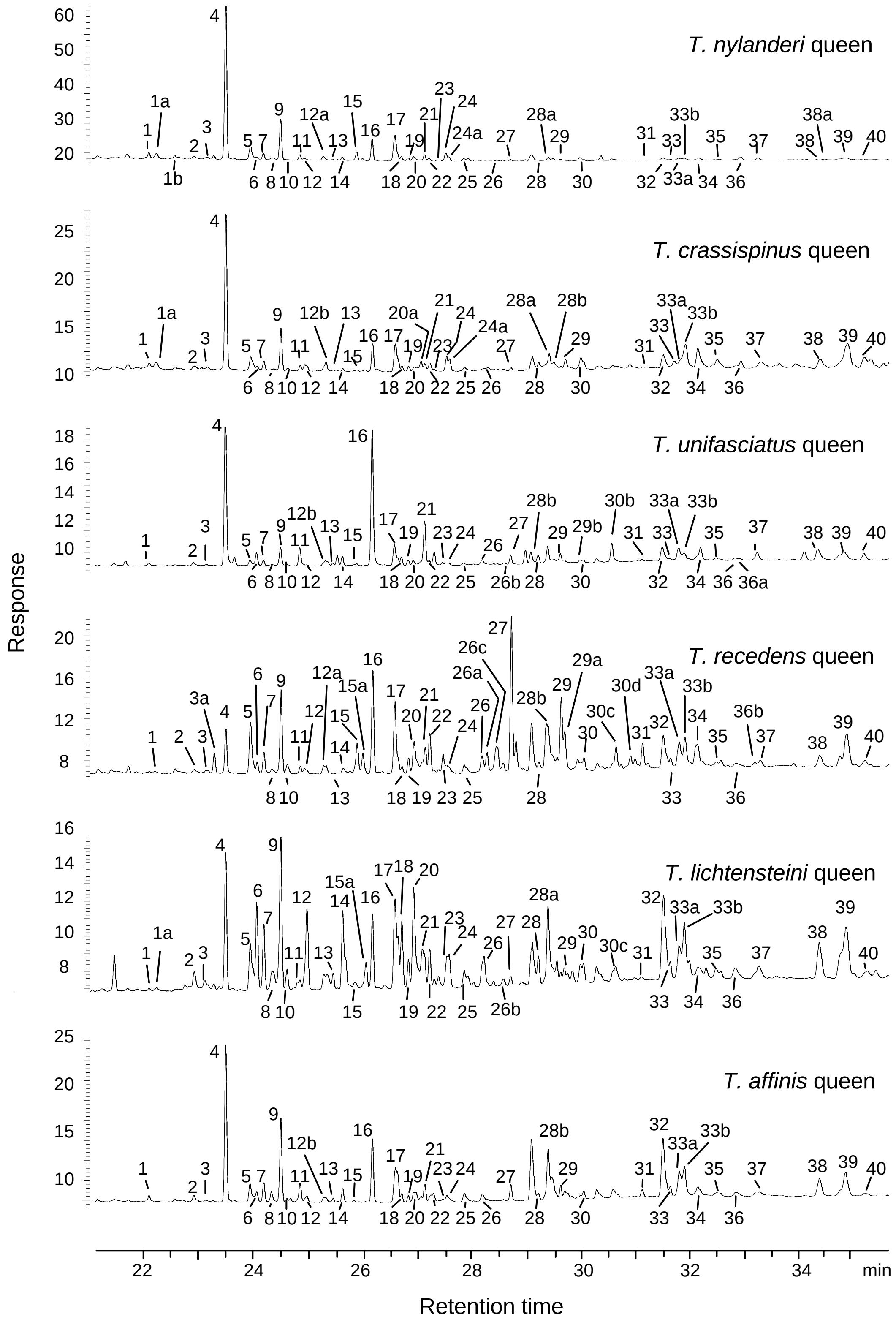

Supplement: Additional file 1 — Gas chromatography profiles. Representative gas chromatography profiles of queens from six Temnothorax species. Peaks used for the statistical analysis are marked with numbers. Identification of peaks is given in Additional file 3. [file 1471-2148-11-55-S1.PDF]
