# Supplementary material for: Queen pheromones in Temnothorax ants: control or honest signal?
Source: BMC Evol Biol. 2011 Mar 1;11:55. doi: 10.1186/1471-2148-11-55 (PMC3060118; doi:10.1186/1471-2148-11-55)

Queen pheromones in *Temnothorax* ants: queen control or honest signal?
Brunner E., Kroiss J., Trindl A. und J. Heinze

Additional file 2 - Peak areas of chemical profiles

Proportions (%) of peak areas in chromatograms from cuticular hydrocarbon extracts of queens and workers in six *Temnothorax* species. Box plots show medians and 25% and 75% quartiles. Whiskers depict the range of 90% of all cases. Extreme outliers are denoted by filled circles. *P*-values of substances differing significantly between the various groups are given in Additional file 3.

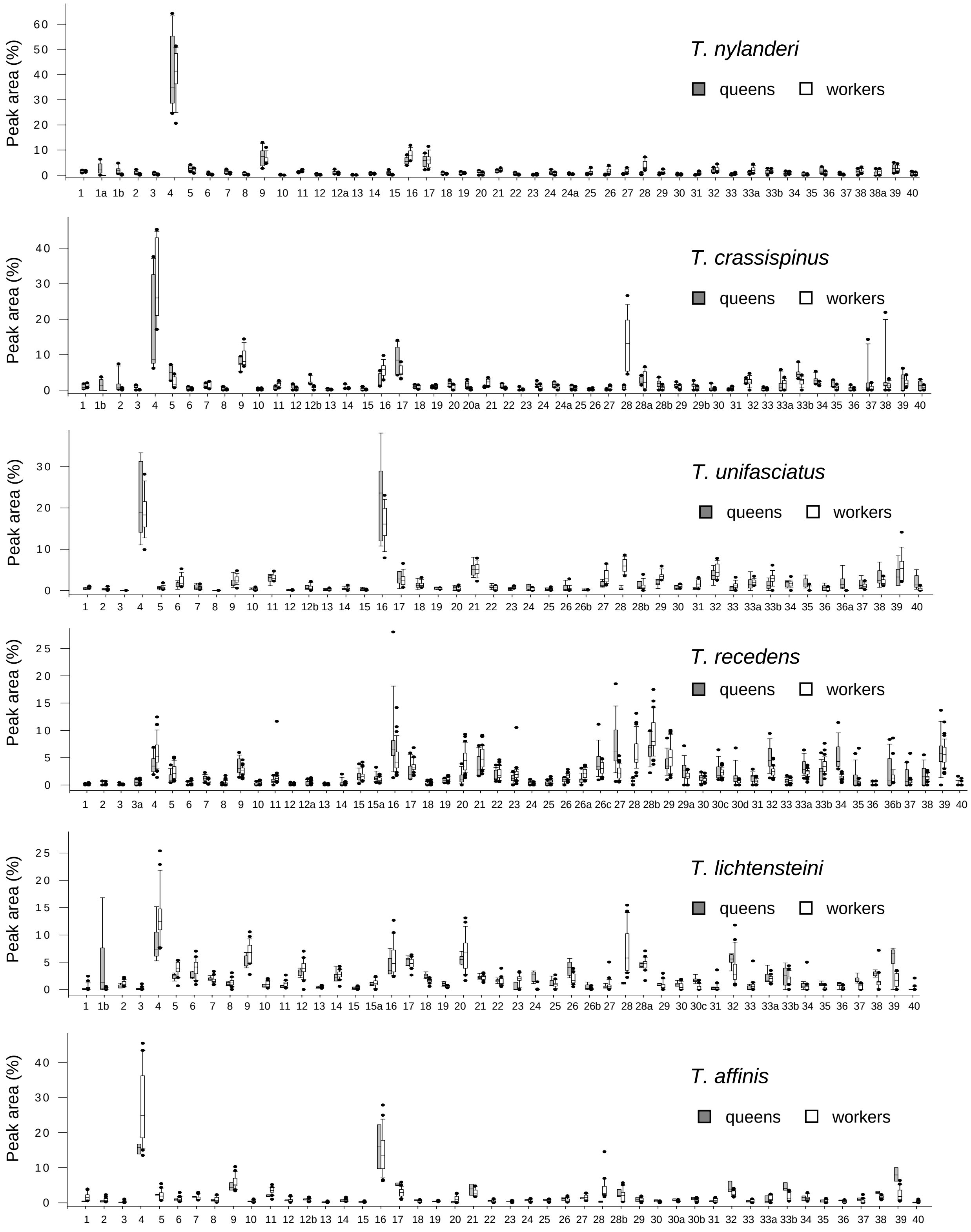

Supplement: Additional file 2 — Proportion of peak areas. Proportions (%) of peak areas in chromatograms from cuticular hydrocarbon extracts of queens and workers in six Temnothorax species. Box plots show medians and 25% and 75% quartiles. Whiskers depict the range of 90% of all cases. Extreme outliers are denoted by circles. P-values of substances differing significantly between the various groups are given in Additional file 3. [file 1471-2148-11-55-S2.PDF]
