# Supplementary material for: Queen pheromones in Temnothorax ants: control or honest signal?
Source: BMC Evol Biol. 2011 Mar 1;11:55. doi: 10.1186/1471-2148-11-55 (PMC3060118; doi:10.1186/1471-2148-11-55)
Supplement: Additional file 3 — Table S1 - Identification of cuticular compounds. Identification of cuticular compounds and differences of their relative amounts between queens (Q) and workers (W) in five Temnothorax species. The number of samples of queens and workers is given in parentheses. Peak numbers correspond with numbers in Additional file 1 and 2. Directions of difference are shown in Additional file 2. Bold p-values from Mann-Whitney U-tests are significant at the 5% probability after Bonferroni's correction (p' < 0.001); n.s = not significant. *Due to very low abundance, peaks marked with a star could not be identified. They had exactly the same retention time in GC as peaks in other species, which could be identified. We therefore assume these compounds to be chemically identical. [file 1471-2148-11-55-S3.PDF]

Queen pheromones in *Temnothorax* ants: queen control or honest signal?
Brunner E., Kroiss J., Trindl A. und J. Heinze

Additional file 3, Table S1– Identification of cuticular compounds

Identification of cuticular compounds and differences of their relative amounts between queens (Q) and workers (W) in six *Temnothorax* species. The number of samples of queens and workers is given in parentheses. Peak numbers correspond with numbers in Additional file 1 and 2. Peak numbers 1-40 consistently appear in all six species, while peak numbers with an additional letter (e.g. 1a) do not appear in all six species. Peaks Directions of difference are shown in Additional file 2. Bold *p*-values from Mann-Whitney U-tests are significant at the 5% probability after Bonferroni's correction ($p' < 0{,}001$); *n.s* = not significant. *Due to very low abundance, peaks marked with a star could not be identified. They had exactly the same retention time in GC as peaks in other species, which could be identified. We therefore assume these compounds to be chemically identical.

Peak No.	Compound	Difference between *T. nylanderi* Q (6) and W (9)	Difference between *T. crassispinus* Q (6) and W (9)	Difference between *T.unifasciatus* Q (5) and W (12)	Difference between *T. recedens* Q(4) and W (18)	Difference between *T. lichtensteini* Q(5) and W (18)	Difference between *T. affinis* Q (10) and W (34)
1	*n*-C_{26}	*n.s.*	*n.s.*	$p < 0.01$	*n.s.*	$p < 0.05$.	$p < 0.01$
1a	unidentified	only in Q	only in Q	-	-	*n.s.*	-
1b	unidentified	$p < 0.01$	-	-	-	-	-
2	4-me C_{26}	$p < 0.05$	*n.s.*	*n.s.*	*n.s.*	$p < 0.05$	*n.s.*
3	3-me C_{26}	$p < 0.01$	$p < 0.05$	$p < 0.05$	$p < 0.05$	*n.s.*	*n.s.*
3a	$C_{27:1}$	-	-	-	*n.s.*	-	-
4	*n*-C_{27}	*n.s.*	*n.s.*	*n.s.*	$p < 0.01$	$p < 0.05$	$p < 0.05$
5	9-me C_{27} +11-me C_{27} +13-me C_{27}	*n.s.*	$p < 0.05$	*n.s.*	$p < 0.05$	$p < 0.05$	*n.s.*
6	7-me C_{27}	$p < 0.05$	*n.s.*	*n.s.*	*n.s.*	*n.s.*	*n.s.*
7	5-me C_{27}	*n.s.*	*n.s.*	*n.s.*	$p < 0.01$	*n.s.*	*n.s.*
8	11,15 di-me C_{27}	$p < 0.05$	$p < 0.05$	$p < 0.05$*	*n.s.*	*n.s.*	*n.s.*
9	3-me C_{27}	*n.s.*	*n.s.*	*n.s.*	*n.s.*	$p < 0.05$	*n.s.*
10	5,x di-me C_{27}	$p < 0.05$	*n.s.*	*n.s.*	$p < 0.05$	*n.s.*	*n.s.*
11	*n*-C_{28}	$p < 0.05$	$p < 0.05$	*n.s.*	$p < 0.05$	*n.s.*	$p < 0.05$
12	3,7 di-me C_{27}	p=0.059	*n.s.*	*n.s.*	*n.s.*	*n.s.*	*n.s.*
12a	x,y di-me C_{28}	*n.s.*	-	-	*n.s.*	-	-
12b	8-me C_{28}	-	$p < 0.01$*	*n.s.*	-	-	*n.s.**
13	6-me C_{28}	*n.s.*	*n.s.*	*n.s.*	$p < 0.05$.	*n.s.*	*n.s.*
14	4-me C_{28}	*n.s.*	*n.s.*	*n.s.*	*n.s.*	*n.s.*	*n.s.*
15	3-me C_{28}	$p < 0.01$	*n.s.*	*n.s.*	*n.s.*	*n.s.*	*n.s.*
15a	unidentified	-	-	-	*n.s.*	*n.s.*	-
16	*n*-C_{29}	$p < 0{,}05$	$p < 0.05$	*n.s.*	*n.s.*	*n.s.*	*n.s.*
17	9-me C_{29} +11-me C_{29} +13-me C_{29} +15-me C_{29}	*n.s.*	*n.s.*	*n.s.*	*n.s.*	*n.s.*	$p < 0.01$
18	7-me C_{29}	*n.s.*	*n.s.*	*n.s.*	*n.s.*	$\mathbf{p < 0.001}$	$p < 0.01$
19	5-me C_{29}	*n.s.*	*n.s.*	*n.s.*	*n.s.*	$p < 0.01$	*n.s.*
20	x,y di-me C_{29}	*n.s.*	$p < 0.05$	*n.s.*	$\mathbf{p < 0.001}$	*n.s.*	$p < 0.05$
20a	unidentified	-	$p < 0.01$	-	-	-	-
21	3-me C_{29}	*n.s.*	$p < 0.01$	*n.s.*	$p < 0.05$	*n.s.*	*n.s.*
22	5,x di-me C_{29}	$p < 0.05$	$p < 0.01$	*n.s.*	*n.s.*	*n.s.*	*n.s.*
23	*n*-C_{30}	$p < 0.05$	*n.s.**	$p < 0.01$	$p < 0.05$	$p < 0.01$	$p < 0.05$
24	3,x di-me C_{29}	$p = 0{,}059$	$p < 0.01$	*n.s.*	*n.s.*	$\mathbf{p < 0.001}$	*n.s.*
24a	3,7 di-me C_{29}	$p < 0.05$	$p < 0.05$	-	-	-	-
25	x-me C_{30}	$p < 0.05$	*n.s.**	*n.s.*	$p < 0.05$	*n.s.**	$p < 0.05$
26	4-me C_{30}	$p < 0.01$*	$p < 0.05$*	*n.s.*	$\mathbf{p < 0.001}$	$p < 0.05$	*n.s.*

Peak No.	Compound	Difference between *T. nylanderi* Q (6) and W (9)	Difference between *T. crassispinus* Q (6) and W (9)	Difference between *T.unifasciatus* Q (5) and W (12)	Difference between *T. recedens* Q(4) and W (18)	Difference between *T. lichtensteini* Q(5) and W (18)	Difference between *T. affinis* Q (10) and W (34)
26a	unidentified	-	-	-	$p < 0.01$	-	-
26b	3-me C_{30}	-	-	n.s.	-	n.s.	-
26c	$C_{31:1}$	-	-	-	n.s.	-	-
27	*n*-C_{31}	$p < 0.01$	$p < 0.01$	$p < 0.05$	**$p < 0.001$**	n.s.	n.s.
28	7-me C_{31}	$p < 0.01$*	$p < 0.01$*	$p < 0.01$	**$p < 0.001$**	**$p < 0.001$**	$p < 0.01$
28a	5-me C_{31}	-	n.s.*	-	-	**n.s.**	-
28b	13,17 di-me C_{31}	-	n.s.*	n.s.	$p < 0.01$	-	n.s.
29	3-me C_{31}	$p < 0.01$	$p < 0.01$	$p < 0.01$	n.s.	n.s.	n.s.
29a	unidentified	-	-	-	$p < 0.05$	-	-
29b	unidentified	-	$p < 0.05$	-	-	-	-
30	3,7 di-me C_{31}	n.s.*	n.s.*	n.s.	n.s.	n.s.	n.s.
30a	x-me C_{32}	-	-	-	-	-	n.s.
30b	4-me C_{32}	-	-	-	-	-	$p < 0.05$
30c	unidentified	-	-	-	n.s.	$p < 0.01$	-
30d	unidentifed	-	-	-	$p < 0.05$	-	-
31	*n*-C_{33}	$p < 0.01$	$p < 0.01$*	$p < 0.01$	$p < 0.05$	n.s.	n.s.
32	9-me C_{33}+11-me C_{33} +13-me C_{33} +15-me C_{33}	n.s.	n.s.	n.s.	**$p < 0.001$**	$p < 0.05$	$p < 0.05$
33	7-me C_{33}	$p < 0.05$	n.s.*	$p < 0.05$	n.s.	n.s.	n.s.
33a	5-me C_{33}	$p < 0.05$	n.s.*	n.s.*	n.s.	n.s.	n.s.*
33b	x,y di-me C_{33}	n.s.	$p < 0.01$	n.s.*	$p < 0.05$	n.s.	$p < 0.05$
34	3-me C_{33}	n.s.	n.s.*	n.s.	**$p < 0.001$**	n.s.	n.s.
35	3,x di-me C_{33}	n.s.*	$p < 0.05$*	$p < 0.01$	n.s.*	$p < 0.01$*	n.s.
36	unidentified	$p < 0.05$	n.s.	n.s.	n.s.	$p < 0.05$	$p < 0.05$
36a	unidentified	-	-	$p < 0.05$	-	-	-
36b	unidentified	-	-	-	n.s.	-	-
37	unidentified	$p < 0.01$	n.s.	n.s.	n.s.	**$p < 0.001$**	n.s.
38	unidentified	n.s.	n.s.	n.s.	n.s.	$p < 0.01$	$p < 0.01$
38a	unidentified	n.s.	-	-	-	-	-
39	unidentified	n.s.	n.s.	n.s.	n.s.	$p < 0.05$	$p < 0.01$
40	unidentified	n.s.	n.s.	$p < 0.05$	n.s.	n.s.	n.s.
