# Supplementary material for: Queen pheromones in Temnothorax ants: control or honest signal?
Source: BMC Evol Biol. 2011 Mar 1;11:55. doi: 10.1186/1471-2148-11-55 (PMC3060118; doi:10.1186/1471-2148-11-55)

**Queen pheromones in *Temnothorax* ants: queen control or honest signal?**
Brunner E., Kroiss J., Trindl A. und J. Heinze

**Additional file 4 – Phylogenetic tree of *Temnothorax* species**
Phylogenetic tree of *Temnothorax* species. Majority rule consensus tree recovered in a Bayesian analysis (4,000,000 generations) with the GTR + I + G model. The tree is based on 651 base pairs of the mitochondrial CO I gene and numbers represent clade credibility values (J. Beibl, pers. comm.).

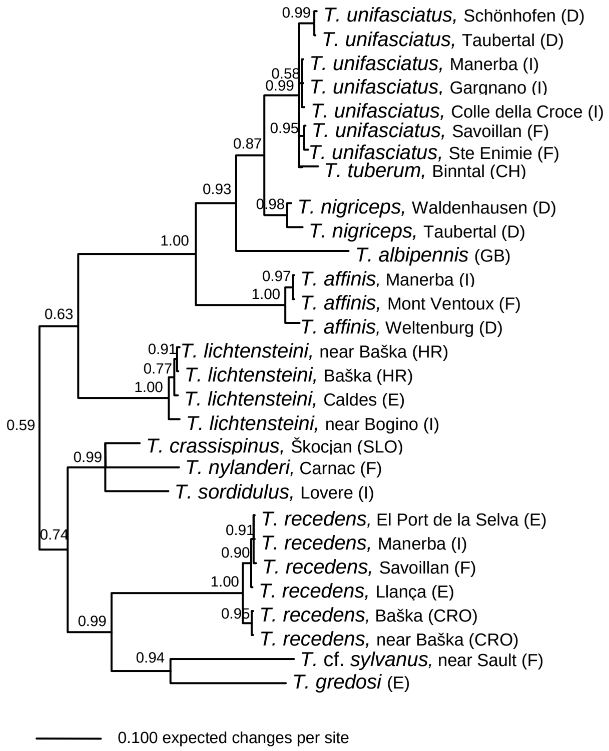

Supplement: Additional file 4 — Phylogenetic tree of Temnothorax species. Phylogenetic tree of Temnothorax species. Majority rule consensus tree recovered in a Bayesian analysis (4,000,000 generations) with the GTR + I + G model. The tree is based on 651 base pairs of the mitochondrial CO I gene and numbers represent clade credibility values (J. Beibl, pers. comm.). [file 1471-2148-11-55-S4.PDF]
